# A Historical Perspective of Influenza A(H1N2) Virus 

**DOI:** 10.3201/eid2001.121848

**Published:** 2014-01

**Authors:** Naomi Komadina, Jodie McVernon, Robert Hall, Karin Leder

**Affiliations:** World Health Organization Collaborating Centre for Reference and Research on Influenza, Melbourne, Victoria, Australia. (N. Komadina);; Monash University, Melbourne (N. Komadina, R. Hall, K. Leder);; The University of Melbourne, Melbourne (J. McVernon);; Victorian Infectious Diseases Reference Laboratory, North Melbourne, Victoria, Australia (J. McVernon);; Victorian Infectious Diseases Services, Melbourne (K. Leder)

**Keywords:** Influenza A virus, viruses, H1N2 subtype, pandemic, swine, reassortant, zoonoses, influenza

## Abstract

The emergence and transition to pandemic status of the influenza A(H1N1)A(H1N1)pdm09) virus in 2009 illustrated the potential for previously circulating human viruses to re-emerge in humans and cause a pandemic after decades of circulating among animals. Within a short time of the initial emergence of A(H1N1)pdm09 virus, novel reassortants were isolated from swine. In late 2011, a variant (v) H3N2 subtype was isolated from humans, and by 2012, the number of persons infected began to increase with limited person-to-person transmission. During 2012 in the United States, an A(H1N2)v virus was transmitted to humans from swine. During the same year, Australia recorded its first H1N2 subtype infection among swine. The A(H3N2)v and A(H1N2)v viruses contained the matrix protein from the A(H1N1)pdm09 virus, raising the possibility of increased transmissibility among humans and underscoring the potential for influenza pandemics of novel swine-origin viruses. We report on the differing histories of A(H1N2) viruses among humans and animals.

## Abstract 2

Emergence and ongoing reassortment of these viruses among animals and humans suggest potential for pandemics.

During 2009, emergence of influenza A(H1N1)pdm09 as a pandemic virus heightened public awareness of the potential for human influenza viruses to mutate. The viruses had been transmitted to animal reservoirs decades earlier, evolved, and were reintroduced to human populations as novel reassortant viruses (*1*). Reinforcing this concept, during 2012, >300 human cases of swine-origin influenza A(H3N2) variant (v) viruses were reported in the United States, predominantly acquired through close contact with pigs at agricultural shows, leading to 11 hospitalizations. The virus had limited person-to-person spread during 2012; a seed vaccine virus was developed for response to the H3N2v strain should the virus become readily transmissible among humans (*2*).

A swine-origin influenza subtype variant, A(H1N2)v, which was lesser known than H3N2v, infected 4 persons attending agricultural shows during the final days of the agricultural show season (*3*). Late emergence of this virus may have limited its spread. Although there was no evidence of human-to-human transmission, there was concern that the presence of the matrix protein derived from the A(H1N1)pdm09 virus, which had been circulating widely in the human population since 2009, could potentially confer the A(H1N2)v virus with increased transmissibility among humans (*3*).

Novel influenza viruses can arise among humans either by direct transmission from mammalian or avian sources or through genetic reassortment. The segmented nature of the influenza viral genome allows reassortment to occur in a host that is simultaneously infected with >2 subtypes of influenza A viruses. Although influenza viruses exhibit some host specificity, swine are susceptible to infection with viruses of avian and mammalian lineages, facilitated by the presence of receptors for both lineages in the respiratory tract. Swine can therefore serve as “mixing vessels” for different lineages, providing an opportunity for novel reassortants to arise. The reassortant viruses may acquire mammalian adaption characteristics, thereby allowing infection of humans to occur (*4*).

A(H1N2) viruses have been described among avian, swine, and human populations. Like A(H3N2) and A(H1N1) viruses, A(H1N2) viruses have become established in swine herds in many regions. In contrast, A(H3N2) and A(H1N1)pdm09 were the only type A viruses documented as circulating among humans as of 2009. Worldwide, 1 case of a human-origin reassortant was reported between 2003 and the events of 2012 in the United States (*5*). Here, we document the distinct lineages of swine and human influenza H1N2 subtypes, cross-species reassortment, and transmission events that result in the emergence of novel viruses.

## Evolution of Influenza A(H1N2) in Swine

Influenza was first recognized as a disease of swine during the 1918 pandemic, when it was observed that families infected with the pandemic virus often saw that their swine herds were also infected (*6*). Although the 1918 A(H1N1) pandemic virus appeared in swine and human populations around the same time, it is not known if the first viruses infected swine and were transmitted to humans, if human and swine populations were infected at the same time, or if the pandemic virus was transmitted from humans to swine (*7*). Once established, the virus evolved along independent evolutionary pathways in both populations (*8*,*9*).

The first A(H1N1) viruses isolated from swine in the United States during 1930 are known as classical swine influenza A(H1N1) viruses (*8*). A(H3N2) viruses were first identified in swine in 1970 during an influenza surveillance study in Taiwan. This study followed the emergence of the A(H3N2) pandemic virus in humans during mid–1968 known as the Hong Kong flu (*10*). Since the initial introduction of human A(H1N1) and A(H3N2) viruses into swine populations, multiple reassortants with differing genetic compositions have arisen (*8*,*9*).

During 1977, large numbers of A(H1N1) viruses were isolated from the swine population of Japan, indicating that the virus had become widespread. These viruses had a high degree of similarity to the classical swine A(H1N1) lineage, and it was postulated that this virus had been imported into Japan by swine from the United States (*11*). After identification of the A(H3N2) virus in humans, this human virus and its variants were isolated from swine in Europe and Asia (*9*). During 1980, Japan reported a period of high incidence of A(H3N2) viruses in swine and high prevalence of the A(H1N1) virus in the swine population, which provided the opportunity for mixed infection to occur; the first A(H1N2) virus reported in swine was in Japan during1980. This reassortant A(H1N2) virus was a classical swine A(H1N1) virus that had gained the neuraminidase (NA) from human A(H3N2) viruses (*11*).

Human influenza viruses were also identified in swine in Europe: the A(H1N1) virus was isolated circa 1938, and A(H3N2) viruses were identified by serologic surveillance of swine during 1968–1970 (*10*). The classical swine A(H1N1) virus was detected in Europe during 1950; however, the virus was not isolated until 1976 (*10*). During 1980, swine A(H1N1) viruses were isolated in France for the first time, and in 1981, A(H3N2) viruses were also isolated from swine. Both subtypes circulated either separately or jointly in the same geographic areas. During 1987 in Brittany, France, when these subtypes were co-circulating among swine in the surrounding region, A(H1N2) viruses were isolated (*12*). These viruses were reassortants of classical swine A(H1N1) and human-like swine A(H3N2) viruses that had circulated since the 1980s (*12*). In 1994, A(H1N2) viruses were isolated from swine in Great Britain for the first time. Unlike the A(H1N2) viruses previously circulating in Asia and Europe, these A(H1N2) viruses appeared to have undergone triple reassortment, inheriting genes from 3 parent sources: the hemagglutinin (HA) from human A(H1N1) viruses that circulated during 1980–1986, the NA from swine A(H3N2) viruses (a reassortant virus), and the avian-like swine A(H1N1) viruses that had emerged in swine in Europe during 1979 (*13*). Whereas the A(H1N2) variants that emerged in France during the 1980s remained localized, the A(H1N2) virus found in the United Kingdom subsequently spread to mainland Europe and became endemic among European swine (*13*,*14*).

In North America, until the late 1990s, influenza in swine was almost exclusively caused by the classical swine A(H1N1)-like virus. Toward the end of the 1990s, initial A(H3N2) viruses were reported (*15*,*16*). These A(H3N2) viruses had 2 genotypes. One reassortant inherited 3 genes (HA, NA, basic polymerase protein [PB]1) from human seasonal H3N2 viruses; the remaining genes originated from the classical swine A(H1N1) viruses. The second genotype was a triple reassortant; its genes originated from human A(H3N2) (HA, NA, PB1), classical swine A(H1N1) matrix, nucleoprotein, and nonstructural (M, NP, NS) genes, and an avian virus (PB2, PA gene); this genotype became the established A(H3N2) swine virus in the United States after its emergence in 1998 (*16*).

In 1999, A(H1N2) virus was initially reported in the United States from pigs in Indiana (*15*). This virus was identified as a second-generation reassortant with the HA from the classical swine A(H1N1) virus and the remainder from the triple reassortant A(H3N2) virus (*15*). In 2005, a second lineage of A(H1N2) viruses was isolated in swine; these viruses had acquired the HA gene from seasonal human A(H1N1) viruses and maintained the triple reassortant A(H3N2) virus genes (*16*).

Shortly after the emergence of A(H1N1)pdm09 virus in the human population, the virus was noted to have re-entered the swine population. In the United States, 9 H1N1/H1N1pdm09 reassortant virus subtypes with various gene constellations were isolated from swine a short time after the first reports of swine infections with the A(H1N1)pdm09 virus. These reassortant viruses contained the matrix gene from the A(H1N1)pdm09 virus. Reassortant H3N2/H1N1pdm09 subtypes containing the matrix gene were also isolated from swine in the United States. In 2010, the first A(H1N2) virus to have reassorted with the with the A(H1N1)pdm09 virus, gaining the A(H1N1)pdm09 matrix gene, was isolated from pigs in Ohio (*17*).

During 2012 in Australia, circulation of novel A(H1N2) reassortant viruses were reported in 2 widely geographically separated swine populations in Queensland, and Western Australia (*18*). Two distinct reassortants were isolated. One, a triple reassortant, contained the HA derived from human A(H1N1) viruses, the NA from human A(H3N2) viruses, and the remainder of genes from the A(H1N1)pdm09 viruses. The triple reassortant viruses were isolated from both pig farms; however, they were distinct from each other and appear to have emerged independently (*18*). The other strain was essentially the A(H1N1)pdm09 virus, which had gained the NA from human A(H3N2) viruses and was isolated only at the Queensland pig farm. The A(H1N2) reassortant viruses circulating in Australia were distinct from A(H1N2) viruses circulating among swine in other countries (*18*). Little is known about influenza viruses in circulation among pigs in Australia because influenza surveillance is not routine; nevertheless, in 2009, A(H1N1)pdm09 viruses were isolated from several pig farms in Australia (*19*). A timeline of the introduction of A(H1N2) viruses into swine is shown in the Figure.

**Figure:**
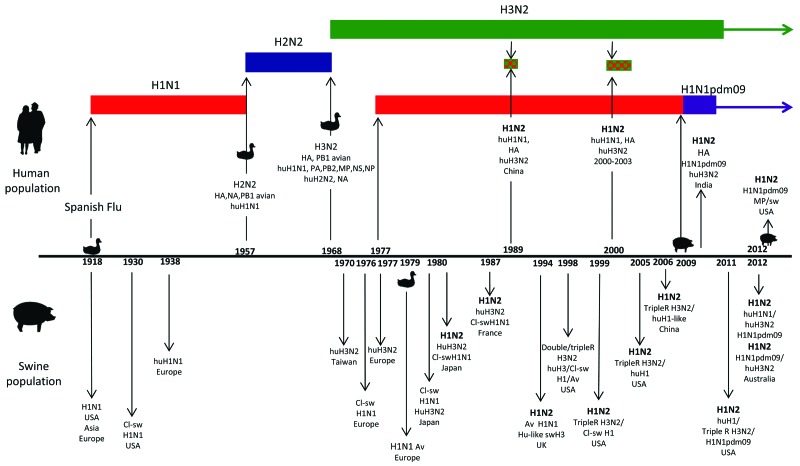
Significant points in the history of influenza viruses that have contributed to the emergence of influenza A(H1N2) viruses in human and swine populations. The bird and swine symbols on the timeline indicate when transmission appeared to occur directly from either avian or swine source into the relevant population. The bird symbols on the 1957 and 1968 time-points indicate that the circulating viruses of the time reassorted with viruses of an avian source resulting in novel subtypes. Significant events leading to the emergence of A(H1N2) in the human population are above the timeline and in swine below the timeline. A(H1N2) viruses appearing in both human and swine populations are indicated in boldface. Genotypes of A(H1N2) emerging in the human population are: 1989 (China), human A(H3N2) virus with hemagglutinin (HA) from human A(H1N1); 2000 (worldwide), same genotype as 1989 virus; 2009 (India), human A(H3N2) virus with HA from A(H1N1pdm09); 2012 (United States), human-like H1, A(H1N1pdm09) matrix, remainder swine H3N2 triple reassortant. Virus origins: Cl-sw, classical swine; hu, human; sw, swine; av, avian; hu-like, human like; double, double reassortant, tripleR, triple reassortant.

A(H1N2) influenza in swine is associated with respiratory illness and can cause sudden unexpected deaths in piglets. Modern farming systems have a higher potential than traditional farming for pig-to-pig transmission of virus to occur, because of the confined operation of intensive pig farms, where crowding results in more frequent and prolonged contact with infected swine. On farms that use traditional farming methods, influenza in swine is a seasonal illness; however, on farms that practice intensive swine farming, swine influenza infections occur year-round, peaking in the colder months (*20*). Influenza usually appears in a herd with the introduction of infected animals, either from movement between farms or by mixing infected pigs with susceptible pigs (*21*). The constant influx of influenza-naive piglets into a herd also contributes to the year-round occurrence of influenza, making the disease difficult to eradicate. Close contact among swine enhances the transmission of virus from swine-to-swine through the nasopharyngeal route by nasal secretions and aerosol droplets. Weather and environmental factors, along with swine husbandry practices of crowding, contribute to the persistence of the virus in the swine herds (*20*,*21*). Influenza has not been isolated from wild boar, although serologic studies have shown that wild boar have been in contact with influenza viruses (*22*). The A(H1N2) viruses, along with H3N2 and H1N1 subtypes, have become enzootic in swine worldwide in areas that have intensive pig farming (*22*).

The availability of large numbers of susceptible piglets and short overall life span of pigs raised for meat production also ensures that the host immune pressure on the virus is less marked in pigs than in humans. Therefore, less antigenic drift occurs in swine influenza viruses than is seen in human viruses (*21*). Currently circulating A(H1N2) swine influenza viruses carry genes originating from humans related to prior reassortment events; however, the decreased immune pressure and slower rate of antigenic evolution in swine could facilitate swine acting as a reservoir for these previously circulating human strains. Populations exposed during the years of prevalence of prior strains would possess long-term immunity to antigenically similar strains, but there may be potential risks to human populations born after the years of prevalence of the older viruses (*22*).

Swine-origin viruses have also infected turkeys, in particular flocks of turkeys that are in close proximity to swine herds (*23*). A(H1N2) virus in turkeys has been associated with respiratory illness and sudden reduction in egg production (*23*). However, unlike in swine, the virus remains a seasonal infection because turkeys are frequently sent to market, which interrupts the infection cycle and prevents the virus from becoming endemic in farmed turkey flocks (*24*). Avian A(H1N2) viruses have also been isolated from wild ducks; however, A(H1) viruses in ducks are considered to be less common (*25*).

## Evolution of Influenza A(H1N2) in Humans

The A(H1N2) viruses isolated from humans during 1989 and 2000–2003 were not of swine origin. Unlike the A(H1N2) viruses, which underwent several reassortment events in swine and became enzootic, the history of A(H1N2) virus in humans differs. The A(H1N1) influenza virus, which emerged in the human population in 1918, was an avian-descended virus, which appeared to have undergone adaptation to humans by unknown mechanisms (*26*). Today, all influenza viruses circulating in the human population carry several gene segments that are direct descendants of the avian-like 1918 A(H1N1) pandemic virus. In 1957, the Asian pandemic virus arose as a result of a reassortment event between the circulating A(H1N1) virus and avian virus to produce a progeny A(H2N2) virus, which retained 5 genes from the A(H1N1) virus and gained 3 novel genes from the avian source. The novel A(H2N2) virus replaced the previous A(H1N1) virus from circulation and continued to circulate until 1968. This new virus then also underwent reassortment, and the 1968 Hong Kong A(H3N2) pandemic virus emerged among the human population (*27*). The new progeny A(H3N2) virus inherited the same 5 genes retained in the 1957 reassortment event. The NA gene was retained from the A(H2N2) reassortment virus, with the HA and PB1 genes gained from an avian source. Descendants of the 1968 A(H3N2) virus continue to circulate in the human population.

In 1977, after a 20-year absence, A(H1N1) influenza virus re-emerged in the human population, causing worldwide epidemics but primarily affecting those under 25 years of age (*28*). This virus, which had ceased circulating in 1957 after the A(H2N2) virus emerged, was antigenically and genetically identical to A(H1N1) viruses that had been isolated in the 1950s (*29*). During the winter of 1978–79, the reappearance of the A(H1N1) virus coincided in some countries with epidemics of A(H3N2), and several instances of co-infection were reported in the United States and Japan. The A(H1N1) and A(H3N2) viruses, and a recombinant A(H3N1) virus, were subsequently isolated and characterized from single samples in both regions (*30*,*31*). Evidence of reassortment between the co-circulating viruses was also found when a reassortant A(H1N1) virus was isolated and found to have HA, NA, the M-gene segment, and NS gene inherited from the A(H1N1) virus and remaining genes from the A(H3N2) subtype (*32*). This virus circulated only in the Northern Hemisphere for 1 season.

Influenza A(H1N1) and A(H3N2) viruses continued to co-circulate among humans, with the predominant circulating subtype changing during following seasons. During 1988, A(H1N2) viruses were reported to be circulating sporadically in China for ≈4 months, although there were no reports of associated influenza outbreaks. This virus was an A(H1N1) and A(H3N2) human influenza reassortant, and it co-circulated with the parent viruses during the winter. There was no reported spread of the A(H1N2) virus to other countries, and after the initial cases in 1989, no further cases were documented in China (*33*).

In 2000, a second reassortant A(H1N2) virus began to circulate; however, this time it did not remain localized but became widespread during the 2001–02 Northern Hemisphere winter. The earliest A(H1N2) viruses isolated were from Thailand during 2000. A small number of A(H1N2) viruses were then detected in Singapore, then Malaysia and Indonesia (2001–02). Two A(H1N2) viruses were isolated in Australia; none were reported from New Zealand or the Pacific region (*34*). In contrast, the emergence of the A(H1N2) virus was associated with substantial outbreaks in the United Kingdom, where it was by far the predominant A(H1) virus during the 2001–02 influenza season. The A(H1N2) virus was first identified in the United Kingdom in September 2001, and it continued to be reported until the end of March 2002. In the United Kingdom, A(H1N2) viruses co-circulated with A(H3N2) viruses in relatively equal proportions, in addition to a small number of A(H1N1) viruses (*35*). Influenza surveillance identified A(H1N2) viruses circulating in Europe and sporadically in Asia, the Middle East, and North and South America (*36*).

A comprehensive study of subjects participating in a vaccine trial conducted in 20 countries on 4 continents identified 65 A(H1N2) viruses from 228 influenza A(H1) viruses isolated (*37*). Most of these A(H1N2) viruses were isolated in South Africa, where A(H1N2) viruses accounted for >90% of the A(H1) cases documented in the trial, which took place during the 2001–2002 influenza season (*37*). Although sporadic circulation of A(H1N2) viruses was reported across the globe, the greatest effect was in the United Kingdom, where A(H1N2) accounted for 54% of the 420 influenza A viruses isolated during the 2001–2002 season (*35*).

The A(H1N2) viruses circulating during 2000–2003 were found to have a similar genetic make-up to those that had circulated sporadically in China during 1988–89 in that they were essentially an A(H3N2) virus where the HA had been replaced with the HA from the A(H1N1) virus (*33*,*38*). The antigenic characterization of these viruses also indicated that the HA of the A(H1N2) viruses were related antigenically to the A(H1N1) virus circulating at the time, including the vaccine strain in use, A/New Caledonia/20/99(H1N1). Further antigenic characterization showed that the NA of the A(H1N2) viruses were closely related to those of the A(H3N2) viruses that were co-circulating (*38*). Genetic characterization indicated that the HA genes of the A(H1N2) viruses were all broadly genetically similar to A/New Caledonia/20/99-like virus, with 2 signature amino acid changes when compared with the A/New Caledonia/20/99 HA gene. Genetic analysis of the remainder of the genome indicated that the other 7 genes were closely related to the H3N2 A/Moscow/10/99-like viruses, which had been circulating in the population at the time of the reassortment event (*35*,*38*). The 2 earliest viruses identified as A(H1N2) from Thailand, however, did not contain these signature changes in the HA gene, suggesting that these A(H1N2) viruses may have arisen from another reassortment event that did not persist (*37*). An A(H1N2) virus from Singapore, isolated from a child who was 3 months of age in October 2000, was the oldest virus of the A(H1N2) viruses containing the signature changes (*34*). Because all the viruses isolated from that point on were found to have <2% divergence, the A(H1N2) viruses were most likely to have originated from a single reassortment. This presumably occurred in 1999 or 2000 in Asia, with the virus subsequently spreading to Europe, the Middle East, Africa, and the Americas (*38*).

In Egypt, Israel, and the United Kingdom, infected persons were mainly 5–15 years of age (*38*), whereas in South Africa, children and the elderly were infected (*37*). In the United Kingdom, which had the greatest number of reported influenza A(H1N2) cases, it is notable that few adults became infected, that only a small number of viruses were isolated from adults >65 years of age, and that 75% of the viruses isolated were from children <15 years of age. Because a similar number of children <15 years of age also became infected with the A(H3N2) virus in the United Kingdom during the same period, it was considered that these children possibly had a primary infection (*35*). Adults and vaccinated persons >60 years of age appeared to have acquired protective immunity to the new subtype, presumably because a substantial proportion of the population had developed immunity either from previous infections by A(H1N1) or A(H3N2) viruses or by vaccination (*35*). The vaccine in use at the time was expected to provide protection against the H1N2 subtype because it contained the H1 from A/New Caledonia/20/99 and the N2 from A/Moscow/10/99-like viruses, which were both genetically and antigenically related to the novel A(H1N2) viruses (*36*).

By early 2003, A(H1N2) viruses were no longer being isolated from human samples. In 2006, an A(H1N2) virus that was a triple reassortant-like virus and, with the exception of the matrix gene, genetically similar to A(H1N2)pdm09 viruses, was isolated from swine in China (*41*). In late 2009, a novel A(H1N2) virus was isolated from a human in India (*5*). This H1N2 virus was a reassortant of A(H1N1)pdm09 and A(H3N2) viruses co-circulating in the population. Although this virus had a similar genetic makeup to previously observed A(H1N2) viruses, the source of the HA component differed and was derived from the A(H1N1)pdm09 virus (*5*). In 2012, swine-origin A(H1N2)v viruses were isolated from 4 humans in the United States. The A(H1N2)v viruses were reassortants of the triple reassortant A(H1N2) viruses viruses circulating in swine in the U.S and A(H1N1)pdm09 viruses (*16*). All persons with cases of A(H1N2)v viruses were linked to the Minnesota state fair and were isolated from humans who had been in close contact with swine. Because these A(H1N2) viruses contained the matrix protein from the A(H1N1)pdm09 virus, there was concern that this virus could transmit more readily in humans (*3*).

## Conclusions

In swine, multiple A(H1N2) virus reassortments have included genetic material from avian, swine, and human influenza viruses and have formed multiple A(H1N2) reassortant viruses with differing genetic compositions over time (*10*). In humans, the A(H1N2) virus has also arisen as a result of the reassortment of human A(H1N1) and A(H3N2) strains, leading to circulation of A(H1N2) viruses that have a similar genetic composition circulating in China in 1989 and worldwide during 2000–2003 (*33*,*34*).

Direct cross-species transfer of swine A(H1N2) is rare and until recently was restricted to reports of single cases from the Philippines (2004) (*39*) and from Michigan and Minnesota in 2007 and 2011, respectively (*3*). Detection of a cluster of 4 swine-origin human A(H1N2)v cases during the final days of the Minnesota agricultural fair in 2012 was therefore a noteworthy event. The rise of H1N2 reassortants containing genes from the H1N1pdm09 virus, in particular the matrix gene, which has been associated with high transmission efficiency (*40*), underscores the fact that influenza reassortment is an ongoing process, that humans can become infected with novel viruses caused by reassortment or transmission of swine origin viruses, and that these novel viruses may have the potential to cause human pandemics.
